# A Synchrotron and Micro-CT Study of the Human Endolymphatic Duct System: Is Meniere's Disease Caused by an Acute Endolymph Backflow?

**DOI:** 10.3389/fsurg.2021.662530

**Published:** 2021-05-31

**Authors:** Hao Li, Gunesh P. Rajan, Jeremy Shaw, Seyed Alireza Rohani, Hanif M. Ladak, Sumit Agrawal, Helge Rask-Andersen

**Affiliations:** ^1^Department of Surgical Sciences, Section of Otolaryngology and Head and Neck Surgery, Uppsala University Hospital, Uppsala, Sweden; ^2^Department of Otolaryngology, Head and Neck Surgery, Luzerner Kantonsspital, Lucerne, Switzerland; ^3^Department of Otolaryngology, Head and Neck Surgery Division of Surgery, Medical School, University of Western Australia, Perth, WA, Australia; ^4^Centre for Microscopy, Characterisation and Analysis, Perth, WA, Australia; ^5^Department of Otolaryngology-Head and Neck Surgery, Western University, London, ON, Canada; ^6^Department of Medical Biophysics and Department of Electrical and Computer Engineering, Western University, London, ON, Canada

**Keywords:** human, synchrotron radiation phase-contrast imaging, reunion duct, Bast's valve, Meniere's disease

## Abstract

**Background:** The etiology of Meniere's disease (MD) and endolymphatic hydrops believed to underlie its symptoms remain unknown. One reason may be the exceptional complexity of the human inner ear, its vulnerability, and surrounding hard bone. The vestibular organ contains an endolymphatic duct system (EDS) bridging the different fluid reservoirs. It may be essential for monitoring hydraulic equilibrium, and a dysregulation may result in distension of the fluid spaces or endolymphatic hydrops.

**Material and Methods:** We studied the EDS using high-resolution synchrotron phase contrast non-invasive imaging (SR-PCI), and micro-computed tomography (micro-CT). Ten fresh human temporal bones underwent SR-PCI. One bone underwent micro-CT after fixation and staining with Lugol's iodine solution (I_2_KI) to increase tissue resolution. Data were processed using volume-rendering software to create 3D reconstructions allowing orthogonal sectioning, cropping, and tissue segmentation.

**Results:** Combined imaging techniques with segmentation and tissue modeling demonstrated the 3D anatomy of the human saccule, utricle, endolymphatic duct, and sac together with connecting pathways. The utricular duct (UD) and utriculo-endolymphatic valve (UEV or Bast's valve) were demonstrated three-dimensionally for the first time. The reunion duct was displayed with micro-CT. It may serve as a safety valve to maintain cochlear endolymph homeostasis under certain conditions.

**Discussion:** The thin reunion duct seems to play a minor role in the exchange of endolymph between the cochlea and vestibule under normal conditions. The saccule wall appears highly flexible, which may explain occult hydrops occasionally preceding symptoms in MD on magnetic resonance imaging (MRI). The design of the UEV and connecting ducts suggests that there is a reciprocal exchange of fluid among the utricle, semicircular canals, and the EDS. Based on the anatomic framework and previous experimental data, we speculate that precipitous vestibular symptoms in MD arise from a sudden increase in endolymph pressure caused by an uncontrolled endolymphatic sac secretion. A rapid rise in UD pressure, mediated along the fairly wide UEV, may underlie the acute vertigo attack, refuting the rupture/K^+^-intoxication theory.

## Introduction

The etiology of Meniere's disease (MD) remains indefinite and continues to foil the afflicted and the medical profession. One reason may be the exceptional complexity of the human labyrinth, its fragile fluid system surrounded by rock-hard bone. There is also a general lack of knowledge about how organs regulate hydrostatic pressure, volume and ion homeostasis (1). The vestibular organ contains separate compartments filled with endolymph fluid. The utricle, semicircular canals, endolymphatic duct (ED), and endolymphatic sac (ES) form a phylogenetically older “pars superior,” while the cochlea and saccule form the “pars inferior” (2). They are interconnected by narrow channels forming an endolymphatic duct system (EDS). The cochlea is linked to the vestibular system via the reunion duct (RD) (3, 4), the saccule to the ED via the saccular duct (SD), and the utricle to the ED via the utricular duct (UD). Consequently, the EDS may allow the exchange of fluid and solutes among the compartments and mediate pressure, thereby regulating fluid homeostasis around the sensory receptors to optimize function.

It is still uncertain if endolymph “circulates” among the partitions, and where it is produced and absorbed, which was initially thought to be confined to each partition. Seymour (2) believed that endolymph was formed in the ES and flowed through the ED to the rest of the labyrinth to insure complete filling of the phylogenetically older utricle and semicircular canals. Yet, experimental investigations suggested a reversed situation, namely, that the lateral cochlear wall secretes endolymph, and a “longitudinal flow” of endolymph exists from the cochlea to the saccule and from there to the ED and ES for reabsorption (5–8). Tracer studies rarely identified a flow toward the utricle (5), supporting Kimura's (9) findings that endolymph is secreted locally in the vestibular labyrinth (9). Endolymph may escape the utricle through the utriculo-endolymphatic valve (UEV) into the UD, ED, and ES (5). A longitudinal flow was later questioned under normal conditions (10, 11), and it was found that endolymph may instead be produced locally and absorbed in the cochlea and utricle (12). Recent molecular analyses show that the ES is essential for the normal development of endolymph volume and absorption, where pendrin, an anion exchange protein encoded by the *SLC26A4* gene, plays an important role during a critical period (13, 14).

The EDS can potentially be disrupted, leading to endolymphatic hydrops (EH), which is believed to underlie the symptoms in MD. Blockage of the RD has been described as a causative factor (15, 16) by some but denied by others (17). Experimental obstruction of the RD results in cochlear hydrops and collapse of the saccule, suggesting that both regions depend on a patent RD (8). Tracer studies indicated that endolymph may circulate from the cochlea through the RD to the ED and ES (5, 18). Collapse of the saccule and maintained utricle filling after RD blockage suggests a lack of endolymph production in the saccule consistent with the absence of an epithelial “dark cell” region or epithelia believed to secrete endolymph (19). Furthermore, experimental blockage of the ES results in EH in guinea pigs, and bony obstruction of the vestibular aqueduct (VA) has been associated with Meniere's disease (7, 17, 20). This suggests that the ES plays an important role in endolymph circulation and reabsorption.

Our knowledge of the anatomy of the human vestibular organ and EDS rests primarily on two-dimensional (2D) histologic sectioning. Reproduction of the three-dimensional (3D) anatomy may enhance our understanding of its function and clarify the pathophysiology of EH and MD. We used high-resolution synchrotron phase contrast imaging (SR-PCI) and micro-computed tomography (micro-CT) to investigate EDS in human temporal bone cadavers. Data were processed using volume-rendering software to create 3D reconstructions allowing orthogonal sectioning, cropping, and tissue segmentation. It revealed for the first time the 3D anatomy of the human UEV and its suspension to the VA. These results may shed new light on the role of the EDS in the etiology and pathophysiology of EH and MD.

## Materials and Methods

### Ethical Statements

#### Human Temporal Bones

Ten fixed adult human cadaveric cochleae were used in this study. Specimens were obtained with permission from the body bequeathal program at Western University, London, Ontario, Canada in accordance with the Anatomy Act of Ontario and Western's Committee for Cadaveric Use in Research (approval No. 06092020). No staining, sectioning, or decalcification was performed on the specimens. Ethics approval for the micro-CT project was obtained from the University of Western Australia (UWA, RA/4/1/5210), and the human temporal bones were provided by the Department of Anatomy at UWA.

#### SR-PCI and Imaging Technique

The SR-PCI technique used in the present investigation was recently described by Elfarnawany et al. (21) and Koch et al. (22). Each sample was scanned using SR-PCI combined with computed tomography (CT) at the Bio-Medical Imaging and Therapy (BMIT) 05ID-2 beamline at the Canadian Light Source, Inc. (CLSI) in Saskatoon, Canada. The imaging field of view was set to 4,000 × 950 pixels corresponding to 36.0 × 8.6 mm, and 3,000 projections over a 180° rotation were acquired per CT scan. CT reconstruction was performed, and the 3D image volume had an isotropic voxel size of 9 μm. The acquisition time to capture all projections per view was ~30 min. For 3D reconstructions of the cochlear anatomy, structures were traced and color-labeled manually on each SR-PCI CT slice (~1,400 slices per sample). The open source medical imaging software, 3D Slicer version 4.10 (www.slicer.org) (23), was used to create detailed 3D representations of the basilar membrane (BM), spiral ganglion and connective dendrites between these structures, which allowed for accurate delineation when compared to traditional 2D slices.

#### Micro-CT

Micro-CT was used to analyze the 3D anatomy of the nerves in the internal acoustic meatus. We used a diffusible iodine-based technique to enhance contrast of soft tissues for diffusible iodine-based contrast-enhanced computed tomography (diceCT). Increased time penetration of Lugol's iodine (aqueous I2KI, 1% I_2_, 2% KI) provided possibilities to visualize between and within soft tissue structures (24). The temporal bone was fixed in a modified Karnovsky's fixative solution of 2.5% glutaraldehyde, 1% paraformaldehyde, 4% sucrose, and 1% dimethyl sulfoxide in 0.13 M of Sorensen's phosphate buffer. Soft tissue contrast was achieved by staining the sample for 14 days, as described by Culling et al. (25). The X-ray micro-CT was conducted using Versa 520 XRM (Zeiss, Pleasanton, CA, USA) running Scout and Scan software (v11.1.5707.17179). Scans were conducted at a voltage of 80 kV and 87 μA, using the LE4 filter under 0.4x optical magnification and a camera binning of 2. Source and detector positions were adjusted to deliver an isotropic voxel size of 23 μm. A total of 2,501 projections were collected over 360°, each with an exposure time of 1 s. Raw projection data were reconstructed using XM Reconstructor software (v10.7.3679.13921; Zeiss) following a standard center shift and beam hardening (0.1) correction. The standard 0.7 kernel size recon filter setting was also used. Images were imported into the 3D Slicer program (Slicer 4.6; www.slicer.org), an open-source software platform for medical image informatics, image processing, and 3D visualization. Images were resized at a scale of 4:1, and opacity and gray scale values were adjusted during volume rendering. The technique allowed reconstruction in three dimensions, and bones were made transparent and cropped.

## Results

**Figure 1:**
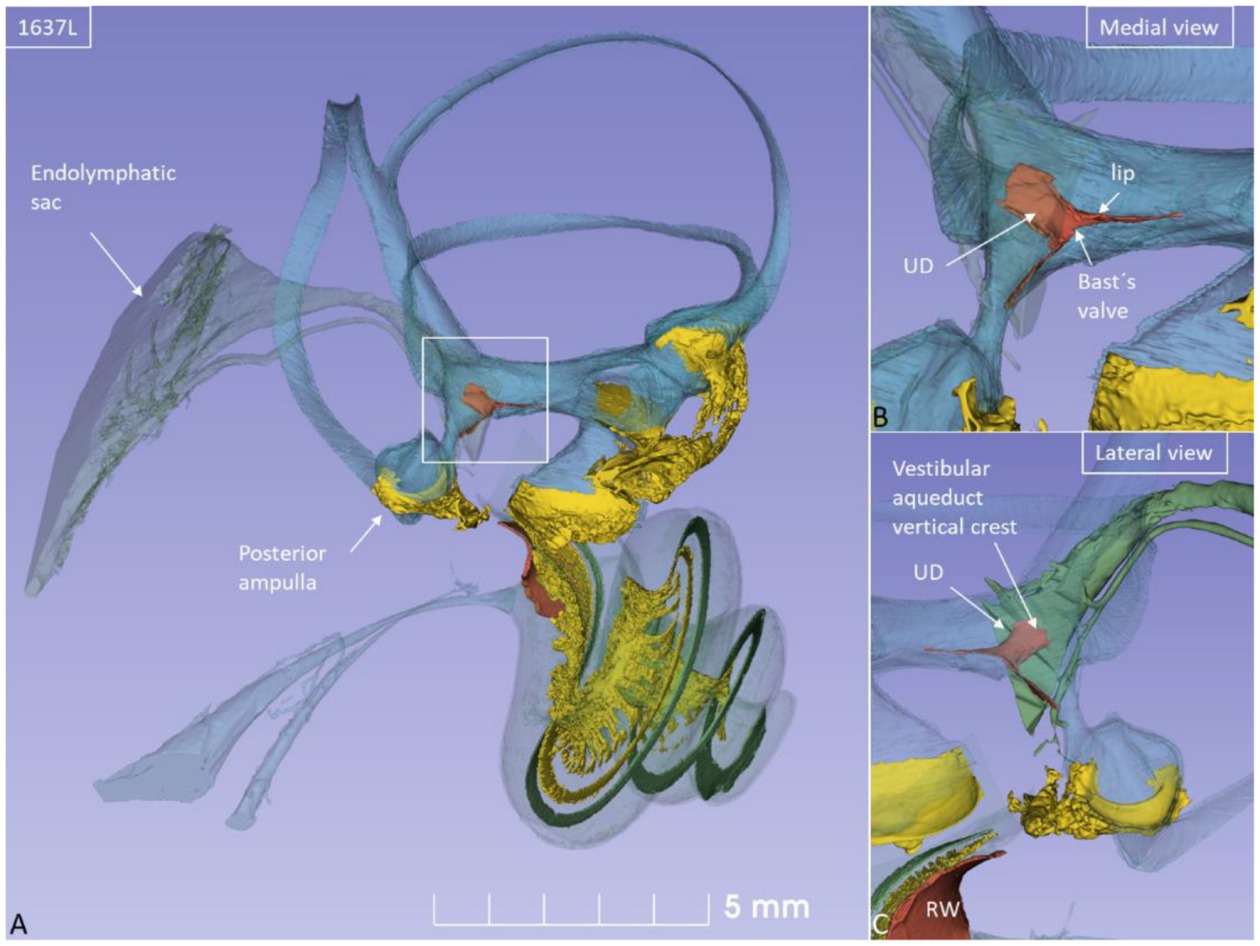
**(A)** SR-PCI of a left human ear with modeling of anatomical details. Maculae and nerve structures are stained yellow. The position of the saccule and utricle and their relationship to the vestibular aqueduct (blue) are shown. **(B)** Medial view shows the position of the UEV relative to the internal aperture of the vestibular aqueduct. **(C)** Posterolateral view.

Non-invasive, high-resolution SR-PCI and micro-CT reproduced both soft and bony tissues of the human cochlea and vestibular organ. Imaging and computer processing provided unique insights into the 3D anatomy of the human vestibular organ with the utricle, saccule, maculae, semicircular canals, and ES (Figure 1). Slicer 3D segmentations and modeling even demonstrated the orientations of the interconnecting ducts of the EDS. Positive and negative contrast enhancement improved micro-CT visualization of soft tissues, while 3D SR-PCI reproduced anatomical details without additional contrast.

**Figure 2:**
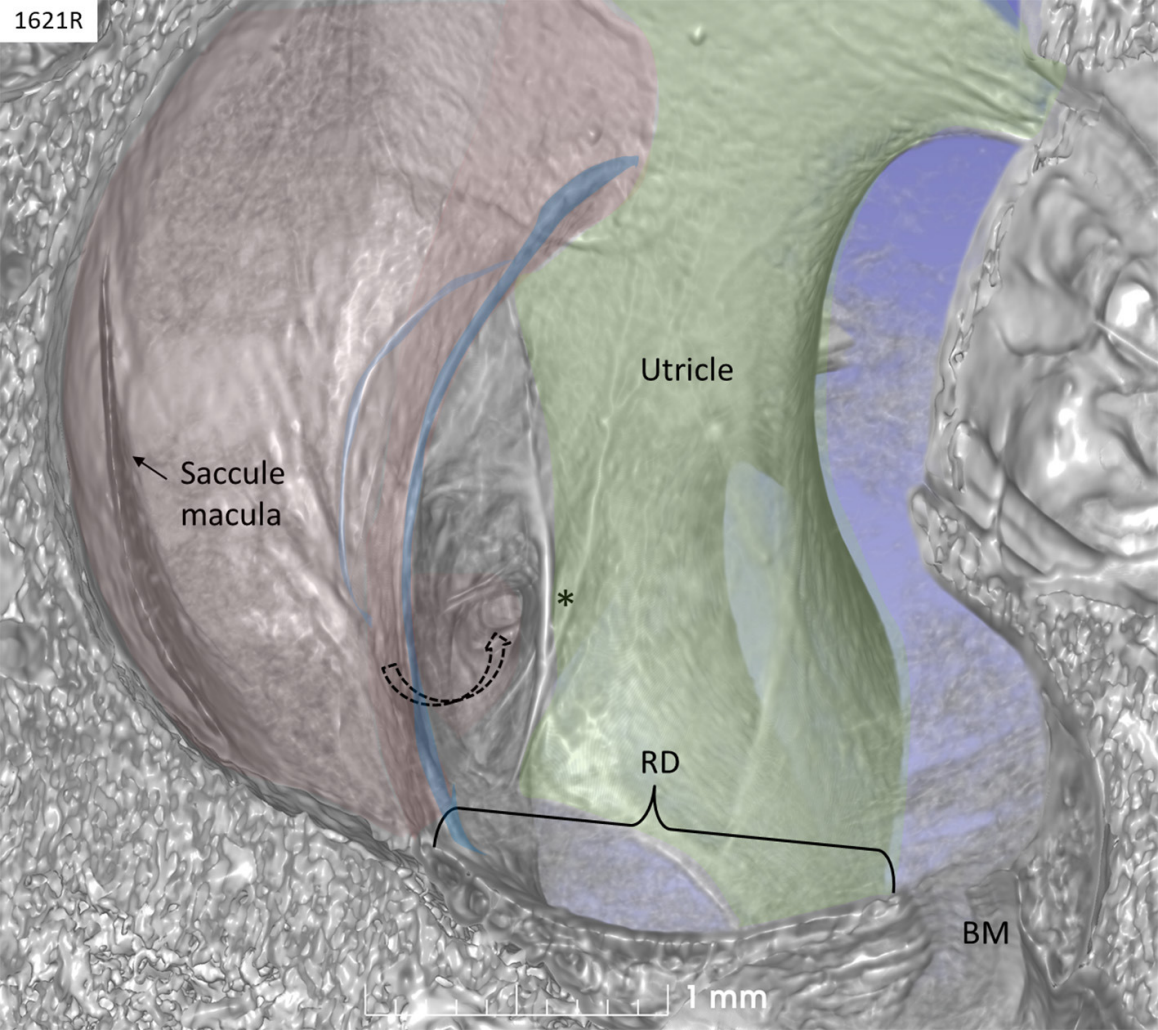
SR-PCI of a left human ear with 3D reconstructions of saccule (red) and utricle (green) (lateral view). The entrance gate to the internal opening of the VA (broken arrow) and the UEV (*) can be seen. The saccule wall contains a reinforced semilunar portion that additionally thickens (blue) against the thinner part. The thinner part and the saccular duct are difficult to discern. BM, basilar membrane. RD, reunion duct.

**Figure 3:**
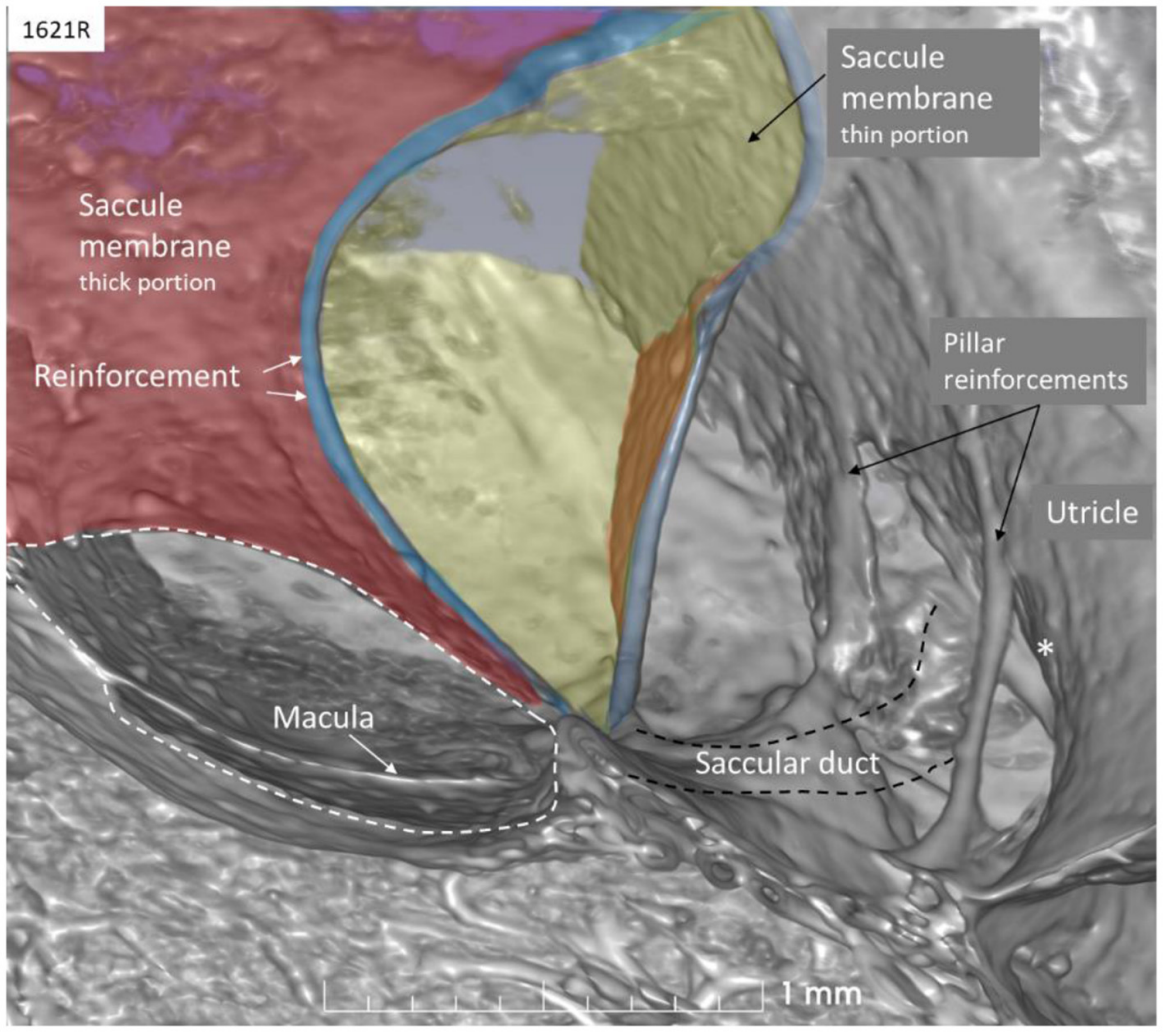
SR-PCI of the saccule (left ear, lateral view). The wall consists of a thick (red) and thin (yellow) part separated by bands of reinforcement (blue). The thin wall appears flaccid. The SD exits near the band and runs against the ED and the UEV (*). The utricle wall is supported by several pillars attached to the internal surface of the surrounding bony wall.

The saccule was bowl-shaped and reached superiorly to the floor of the utricle to which it partially adhered. The saccule macula was placed in a pit in the medial bony wall margined posteriorly by a lip of the spherical recess. The saccular wall was thicker near the macula and had a thinner triangular-shaped part facing the middle ear (Figures 2, 3).

The thicker part contained a fibrous stratum between the mesothelial and epithelial layers. It also thickened into marginal bands where the thick portion transitioned into the thinner portion. These bands reinforced the junction between the saccule and utricle. The saccule narrowed basally into a flat funnel-shaped opening to the RD. The SD exited posteriorly at the spherical recess and ran along the bony wall to the ED and the VA. Its direction was perpendicular to the RD. The thin-walled SD was sometimes difficult to visualize in 3D, but could occasionally be segmented from individual sections. The utricle lay horizontal with the macula slanting from vertical to horizontal at the nerve entry.

**Figure 4:**
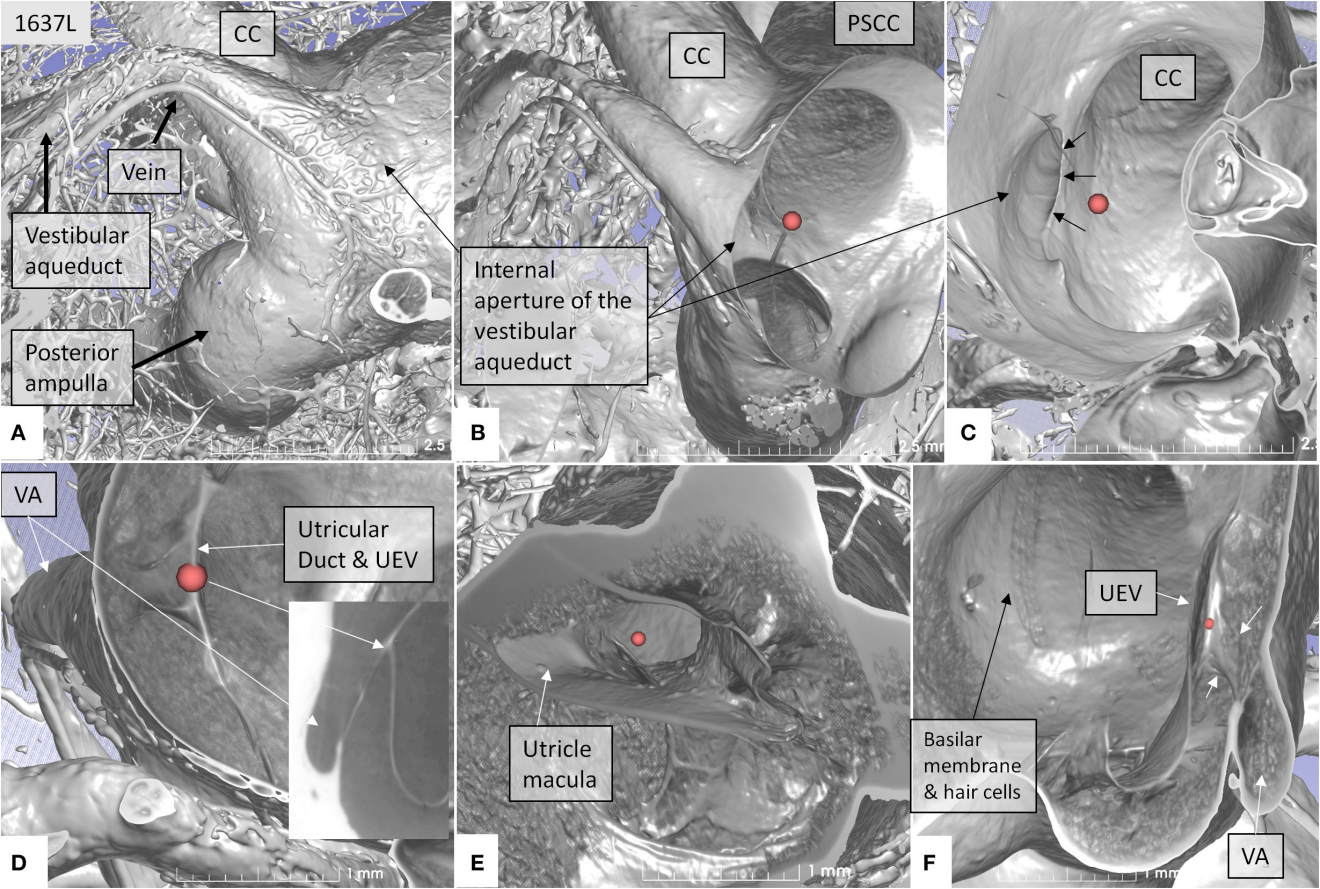
SR-PCI showing the position of the UEV relative to the internal opening of the VA in a left bone. **(A)** Medial view shows the entrance of the VA into the vestibule. A vascular plexus surrounds the VA that drains into the vein of the VA. **(B)** Anterior cropping shows the VA and the position of the UEV (red fiducial). **(C)** Bony algorithm shows the internal opening of the VA relative to the UEV (red fiducial). **(D)** Adjusting opacity gradient reveals both the UEV and the UD (red fiducial). **(E)** Lateral view shows the position of the utricle macula and the UEV (red fiducial). **(F)** Superior view and modification of gradient opacity shows the UD (white arrows) and the UEV (red fiducial). The BM and the rows of hair cells are seen. CC, common crus; UEV, utriculo-endolymphatic valve; VA, vestibular aqueduct; PSSC, posterior semicircular canal; UD, utricular duct; BM, basilar membrane.

**Figure 5:**
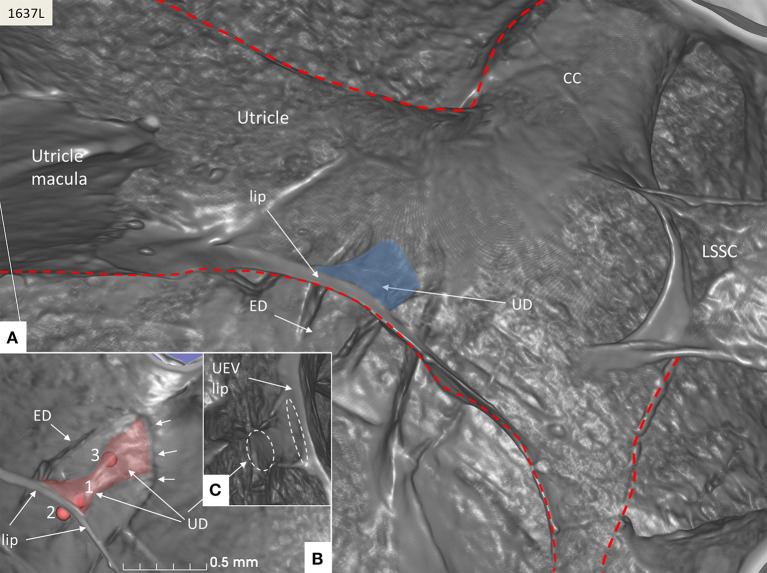
SR-PCI of the UD (blue) at the internal aperture of the VA. **(A)** UEV lip (Bast's valve) is located near the floor of the utricle. The broken red line delineates parts of the vestibular labyrinth. **(B)** Lateral view shows the UD and the ED at the opening of the VA (small arrows). Fiducials (red) mark (1) the position of the mid-portion of the UEV, (2) where the UD reaches the base of the lip, and (3) at the division of the UD and ED. **(C)** Image shows the lateral disc running from the vertical crest of the VA to the UEV. The broken white lines depict the lumen of the UD. LSSC; lateral semicircular canal.

**Figure 6:**
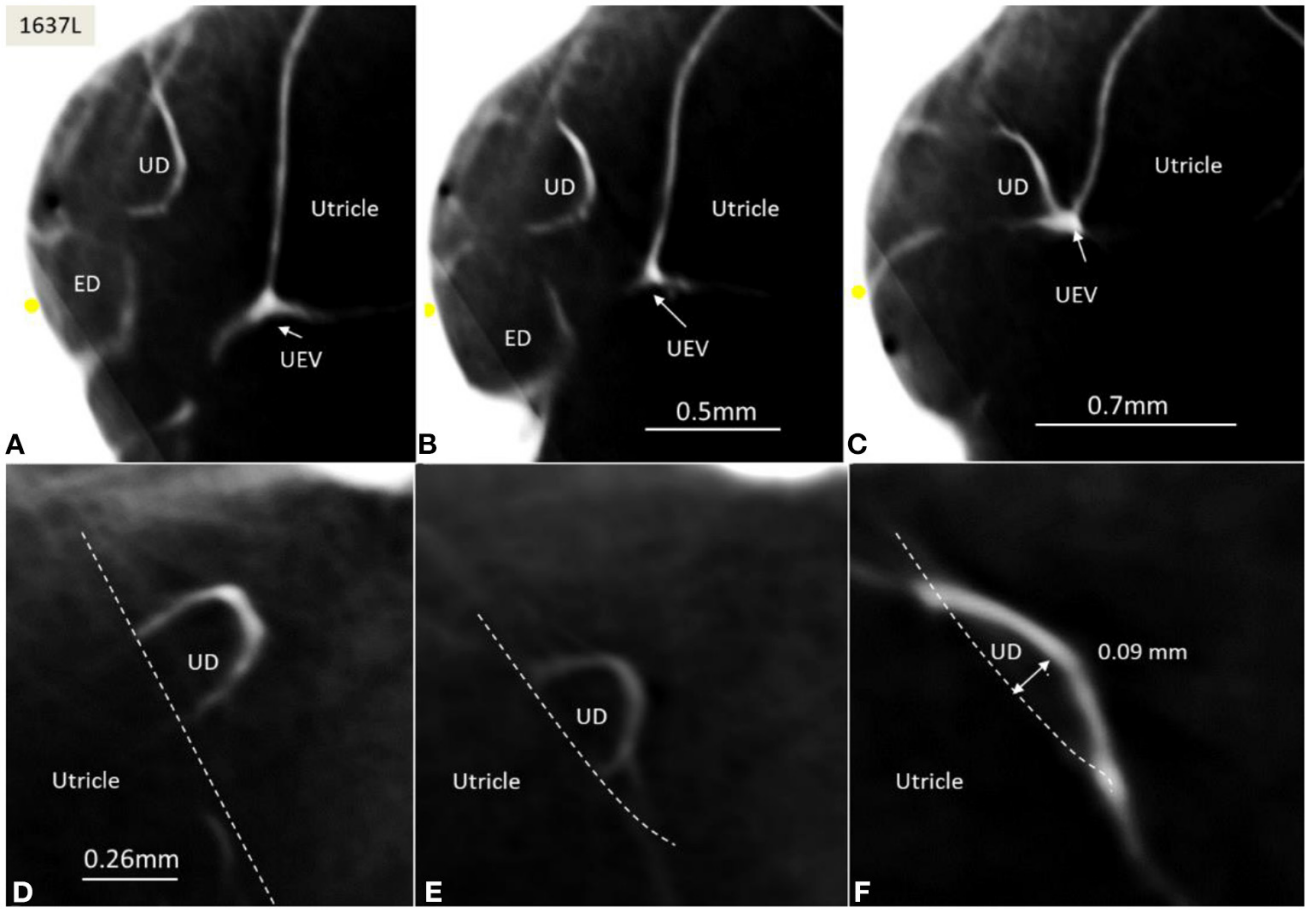
**(A–C)** SR-PCI of the UD and ED at the internal aperture of the VA. The UEV is closed and connected with a membranous strand against the bony wall. **(D–F)** Lateral view shows serial sections of the UD running against the UEV. Its lumen narrows against the valve. The diameter of the UD in **(D)** is 0.32 mm.

**Figure 7:**
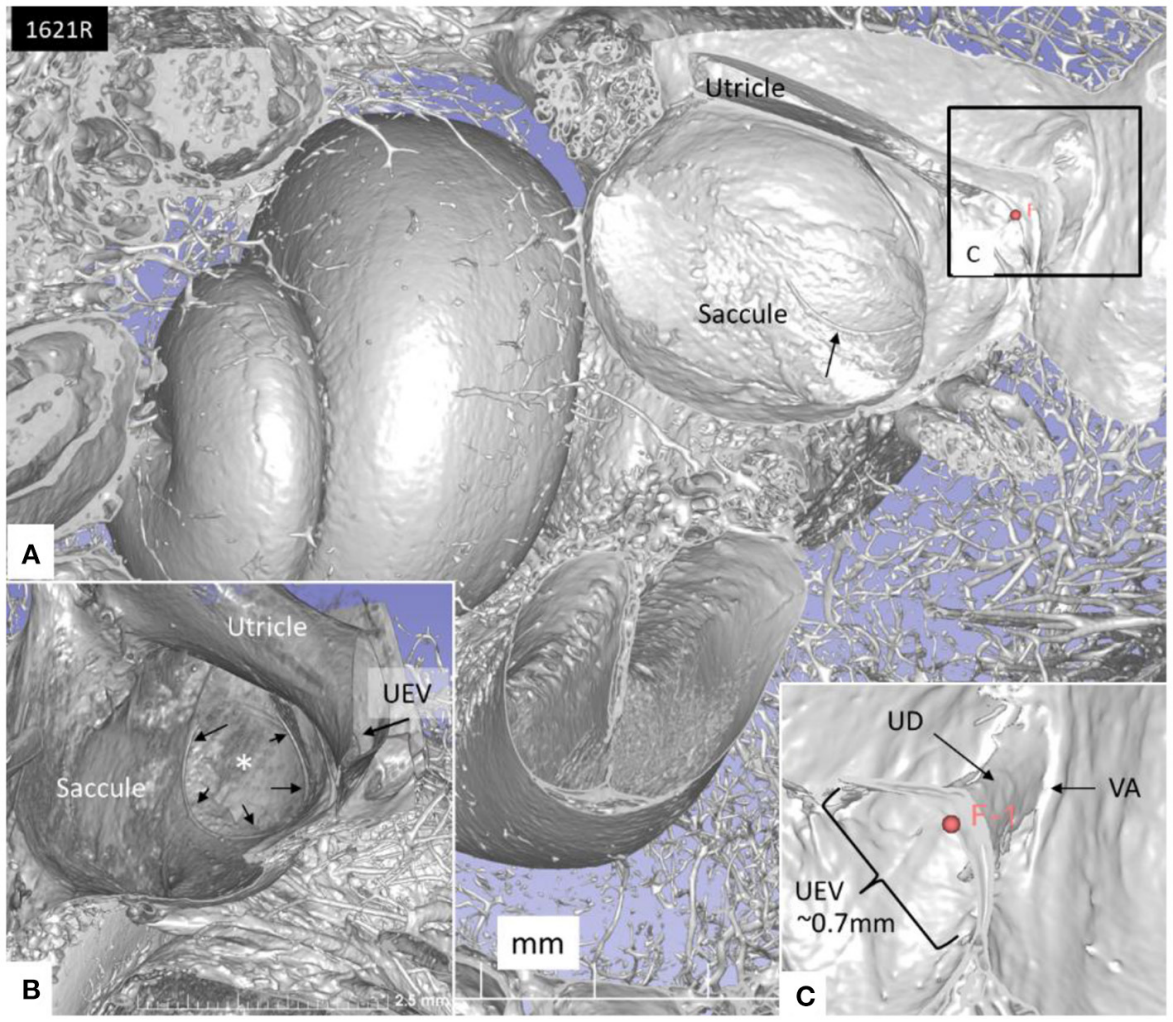
**(A)** SR-PCI of the left cochlea and vestibule sectioned and viewed laterally. **(B)** The saccule wall consist of a thicker part (small black arrows) and a thinner part (*). The UEV is located in the posterior-inferior part of the utricle (arrow). **(C)** The semilunar-shaped opening of the UEV is shown in higher magnification and is marked with a red fiducial. VA, vestibular aqueduct.

**Figure 8:**
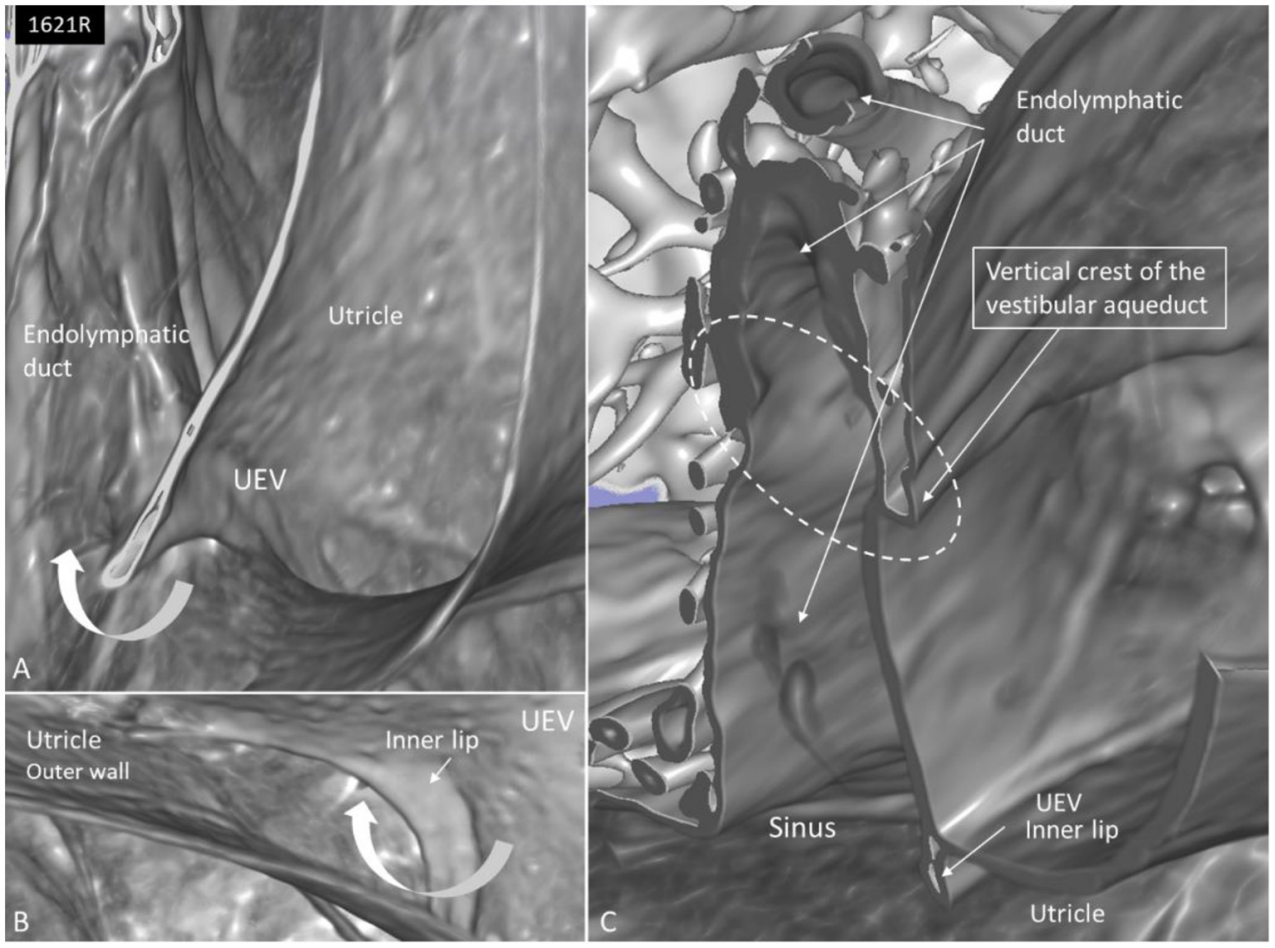
**(A)** SR-PCI and higher magnification of the UEV and the slit-like opening in the utricle. **(B)** The outer utricle wall is thin but can be seen reaching the inner lip of the valve. **(C)** Coronary section shows the internal opening of the vestibular aqueduct (circle, broken lines). A membrane disc connects the vertical crest with the UEV. The epithelial wall of the UD is not visualized. The endolymphatic duct is surrounded by several blood vessels. Sinus: sinus portion of the endolymphatic duct.

**Figure 9:**
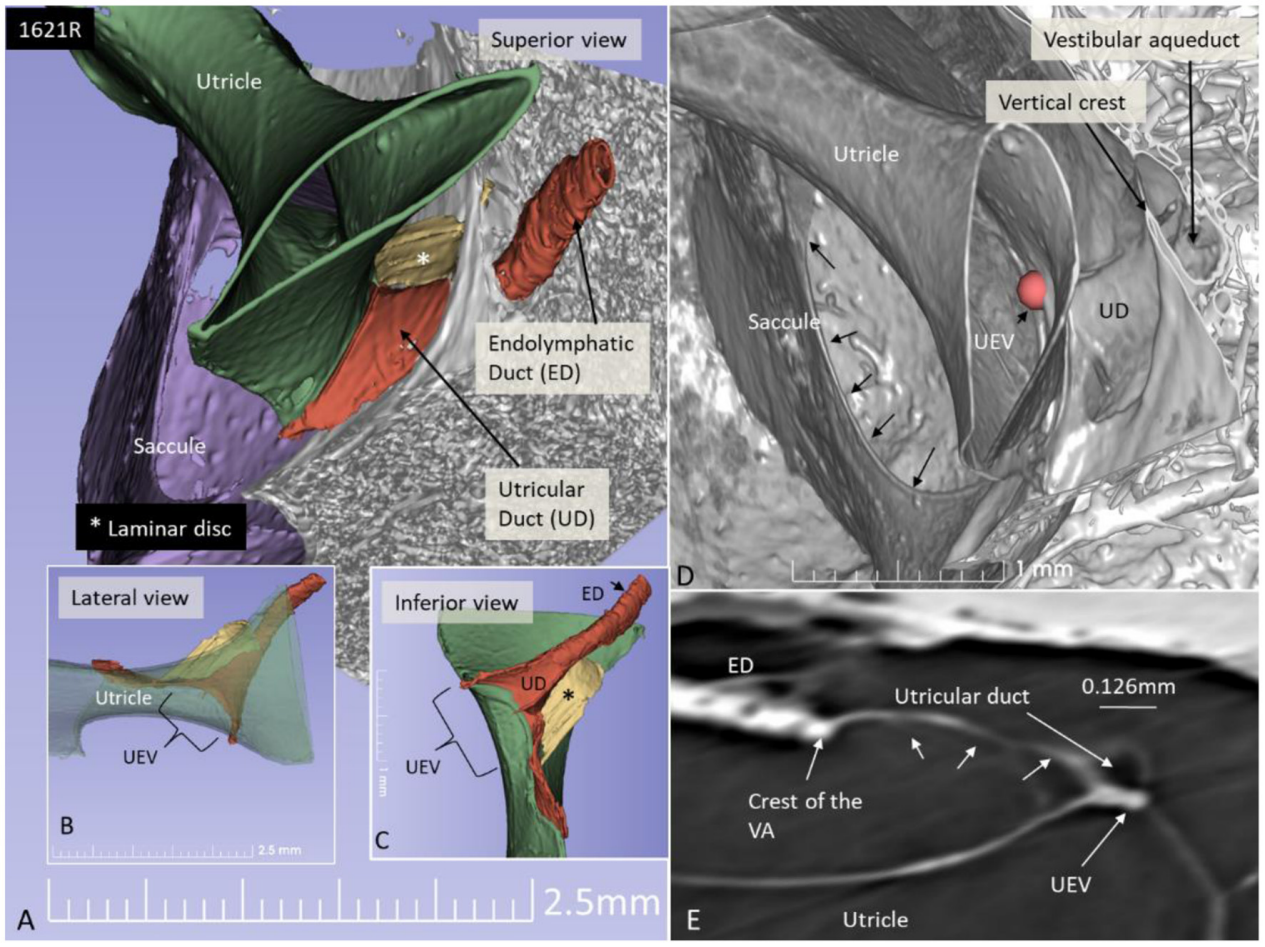
SR-PCI 3D reconstruction and model of the posterior part of the vestibule in a left ear (superior view). **(A)** The endolymphatic duct (ED) and utricular duct (UD) can be seen. A laminar disc (*) extends from the vertical crest of the internal opening of the vestibular aqueduct (VA) to the utricle. It also covers the UD. **(B)** Lateral view of the modeled UEV is viewed through a partly transparent utricle. **(C)** Modeled UEV is viewed from inferior. **(D)** UEV is shown from inside the utricle (red fiducial). The UD wall is reinforced by the laminar disc. **(E)** Horizontal section shows the UD and UEV. The laminar disc (small arrows) runs from the vertical crest to the UEV. It keeps the UD closely associated with the UEV. Note that the UD passes on the UEV allowing increased pressure in the UD to be transmitted and may push the valve to open.

The rather wide UD separated from the ED soon after its entry into the labyrinth and reached the inferior part of the posteromedial wall of the utricle (Figure 4). The UD and UEV are viewed in Figures 5–**9**.

The UD lumen changed from round to ovoid and slit-like (Figures 5, 6). The round portion had a diameter of 0.26 × 0.24 mm in specimen 1637L, where it was best viewed. It flattened and narrowed against the UEV. The division between the UD and ED was located 0.2–0.4 mm from the internal aperture of the VA. The UEV was identified at ~0.7 mm from the vertical crest of the VA (Figure 4). This distance varied, and in one specimen, the distance was 1.6 mm (2R). The valve was crescent-shaped with a superior, thicker lip attached to the inner utricle wall (Figures 8, 9). The lip was suspended by a thickened reinforcement in the utricle wall (Figures 1, 4, 5, 9). This support extended as a ligament-like suspension to the medial bony wall together with fibrous pillars. A suspensory disc stretched from the vertical crest of the VA to the lip of the valve. It was also associated with the lateral wall of the UD (Figures 5C, 9C). Additional fibrous pillars connected the utricle wall with the surface of the bony wall. The mean width of the UEV was 0.69 mm (0.68, 0.65, and 0.74 mm in three measurable specimens). Several blood vessels followed the medial wall of the intra-labyrinthine ED against the VA (Figure 6C).

**Figure 10:**
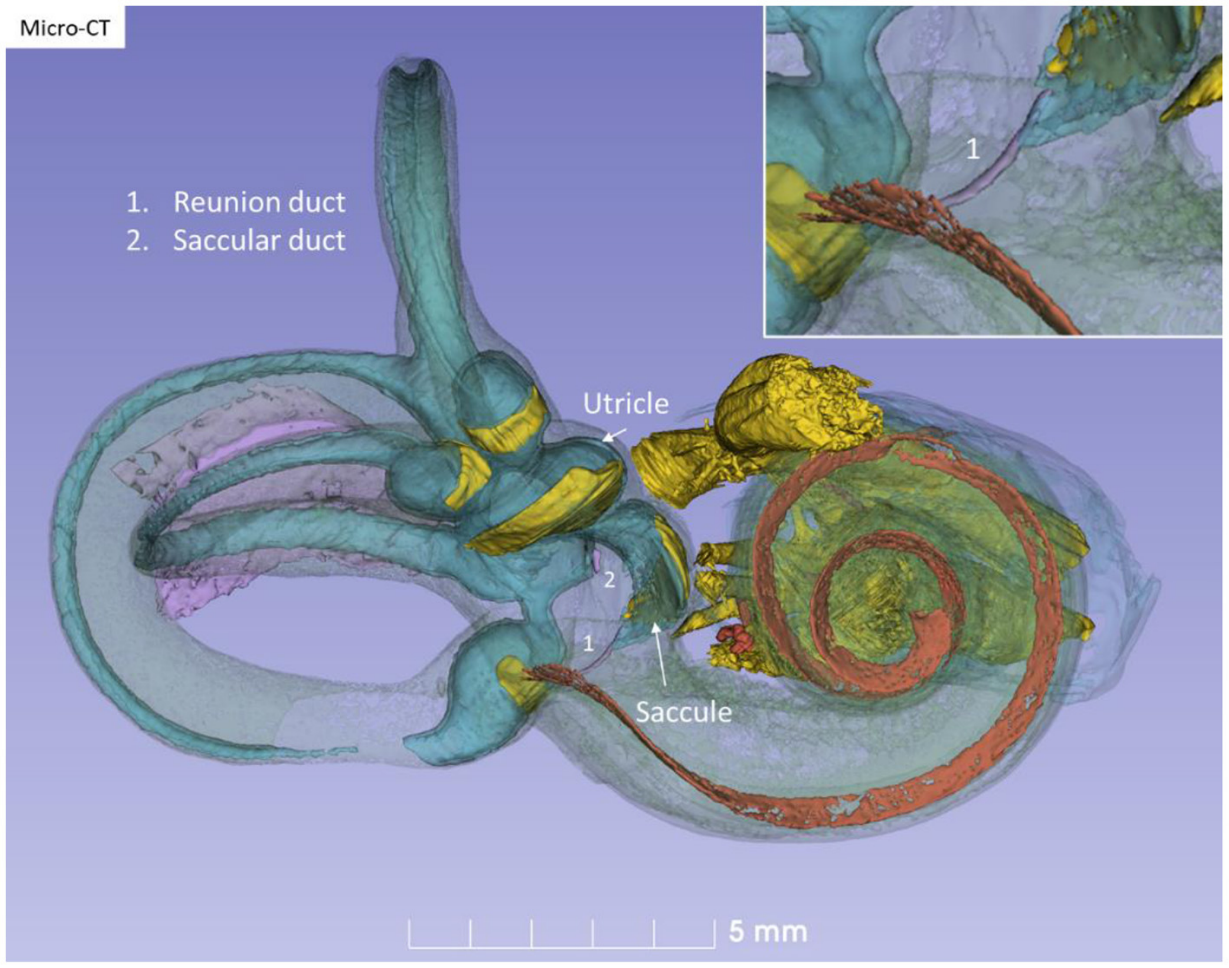
Micro-CT and 3D modeling of a right human temporal bone (Stenver's view). The membrane labyrinth is shown in different colors after the bony capsule is made semi-transparent. The vestibular neuro-epithelium and nerves are yellow. The basilar membrane is colored red. The inset shows RD (1).

**Figure 11:**
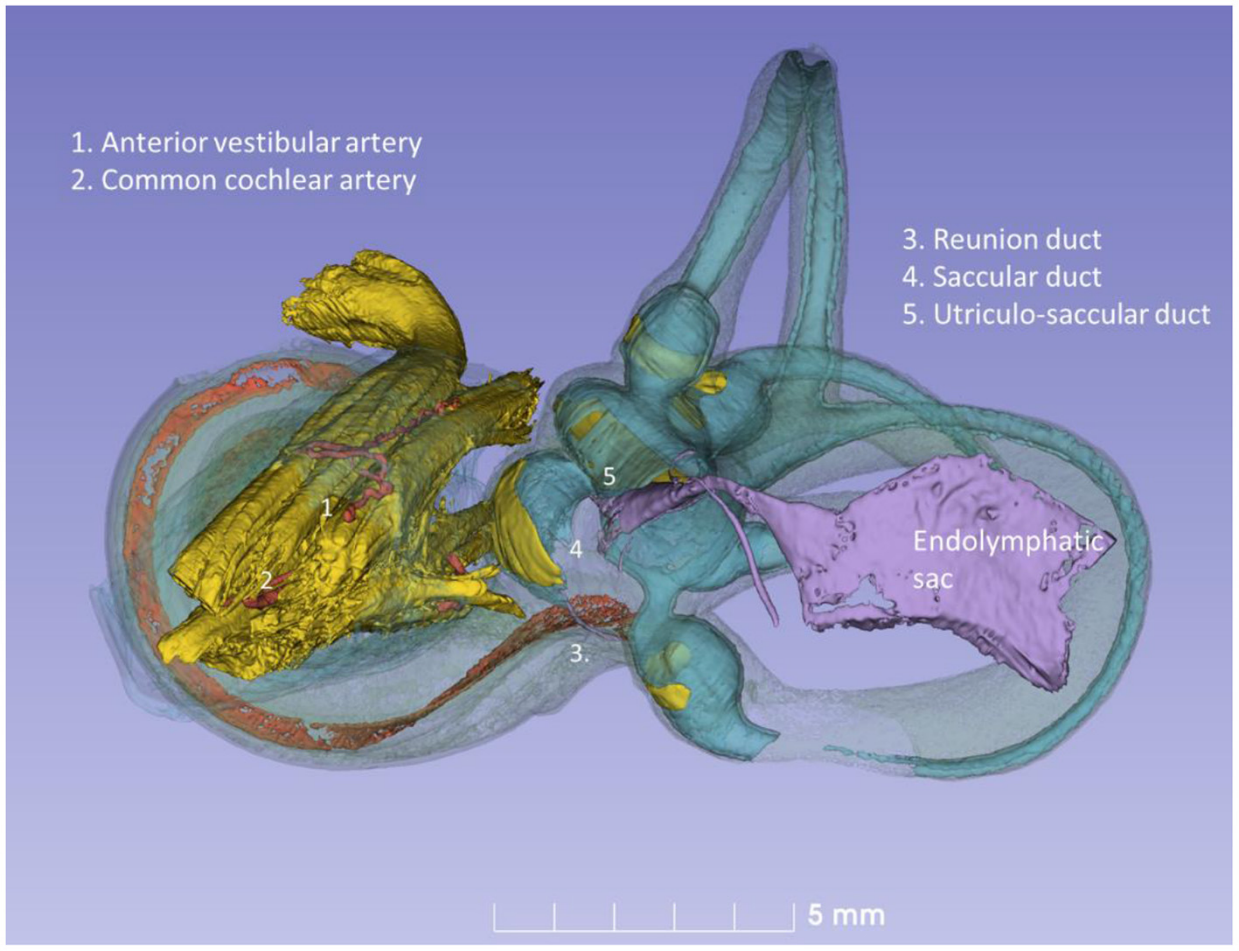
Posterior view of the modeled inner ear shown in Figure 10. The position of the saccular and utricular ducts are visualized. The internal auditory canal is shown with cranial nerves and arterial blood vessels supplying the inner ear.

**Figure 12:**
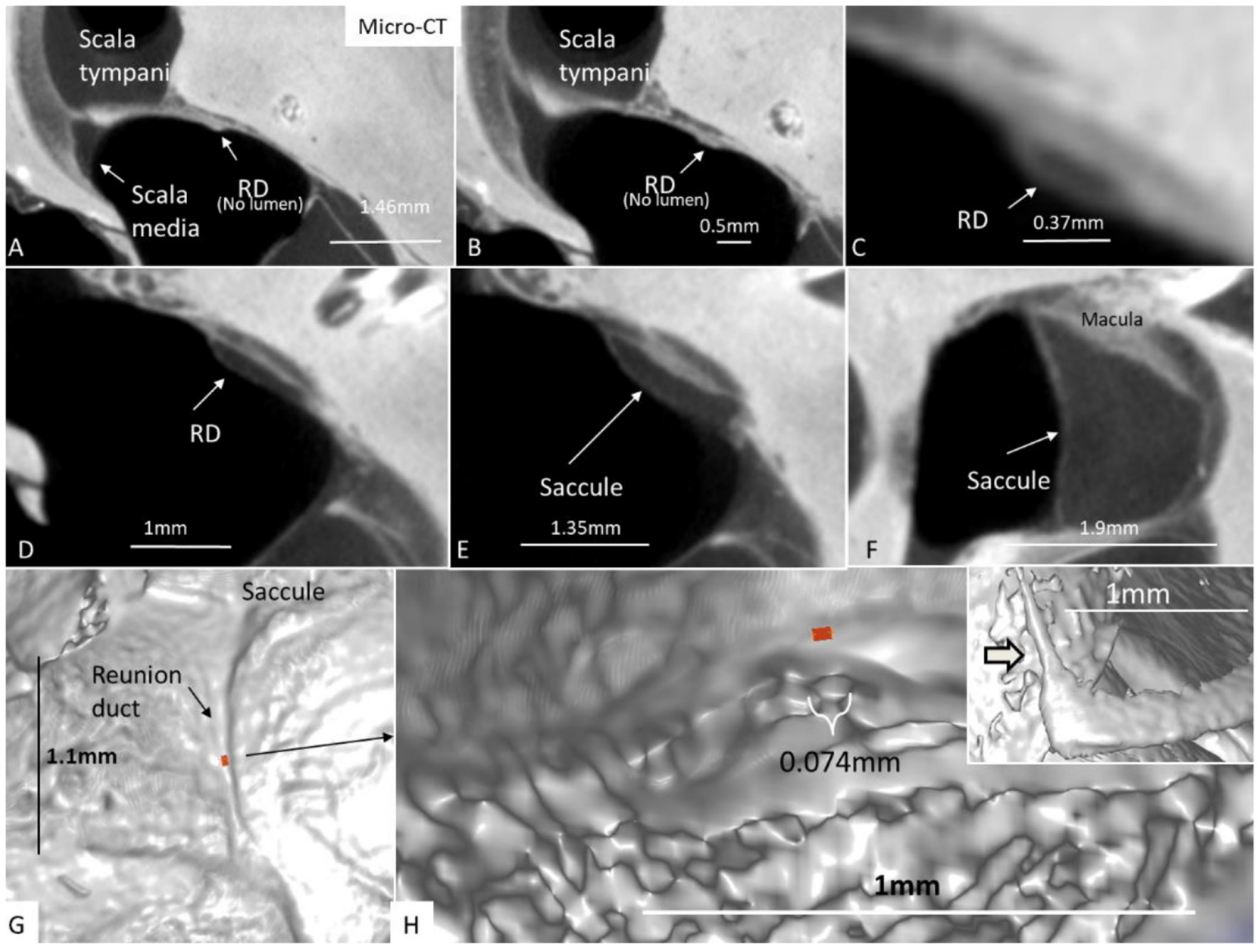
**(A–F)** Micro-CT and serial sections show the reunion duct (RD) from the saccule to the cochlear duct. **(G)** Surface view of the RD. Its mid-portion is cropped and shown in **(H)** The RD diameter is less than a 10th of a millimeter. The inset in H shows the angle formed between the RD and the vestibular end of the cochlear duct.

**Figure 13:**
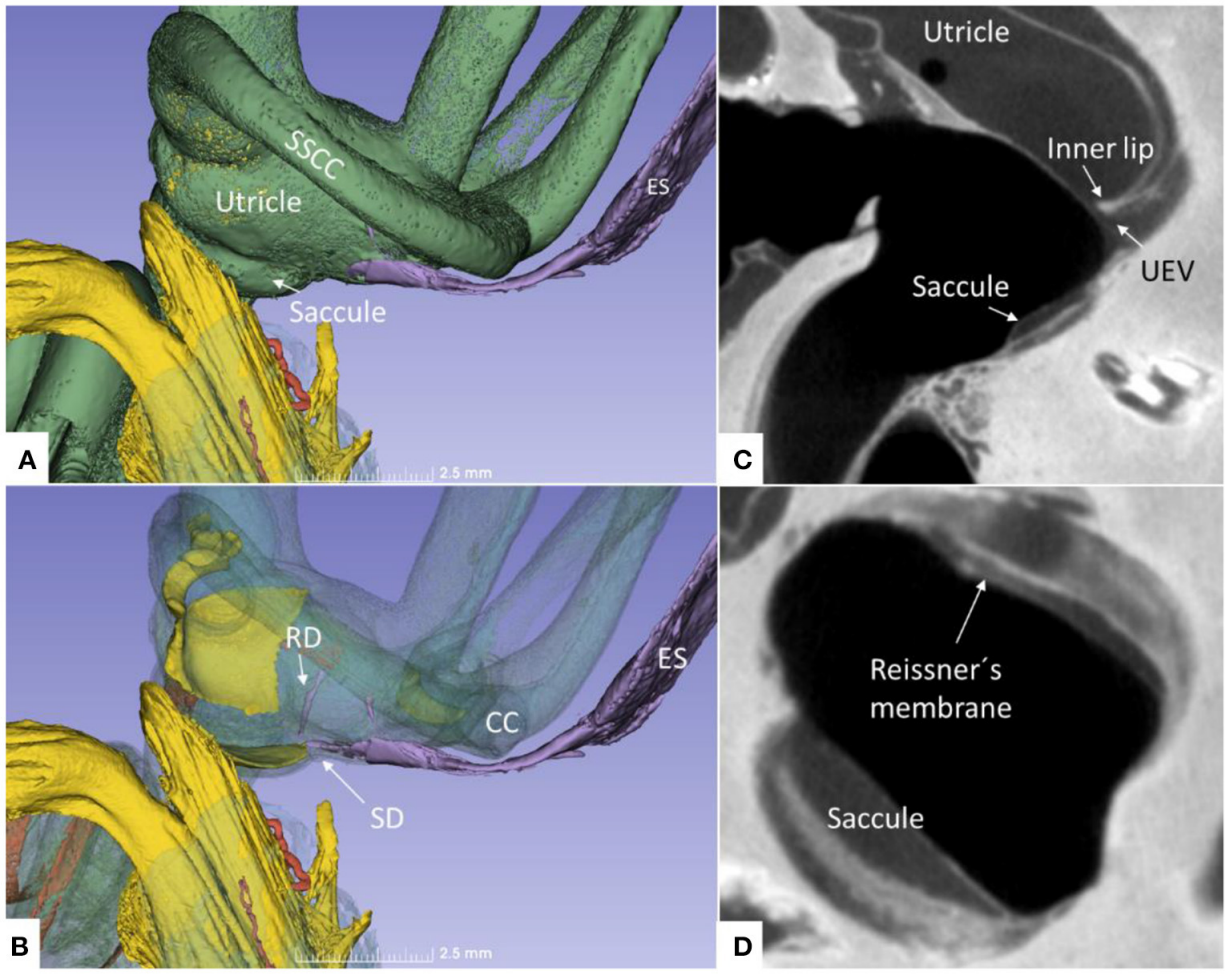
**(A)** Superior close-up view of micro-CT shown in Figure 11. The internal aperture of the vestibular aqueduct (lilac) is seen near the common crus (CC) and the SSCC. **(B)** The saccular and reunion ducts are visible after making the bony capsule transparent. The RD runs almost perpendicular against the saccular duct. **(C)** Horizontal section of the UEV. **(D)** The saccule and cochlear endolymphatic space at the *cecum vestibulare* are shown. SSCC, superior semicircular canal; SD, saccular duct; RD, reunion duct; ES, endolymphatic sac.

The RD was narrow and generally difficult to follow. The RD exited the caudal region of the saccule. It was best viewed with micro-CT and ran from the basal funnel of the saccule against the cochlea along the bony wall. Its smallest diameter was < 0.1 mm (Figures 10–12). It was positioned on a shallow increase of connective tissue on the spiral lamina wall and was best visualized with micro-CT since air had entered the vestibule, thus enhancing contrast. A vestibular overview show an hourglass-shaped fold (Figure 12G). Figures 12A–F shows serial micro-CT sections of the RD from the saccule to the scala media. At some points, the RD seemed collapsed. In Figures 12G,H, the cross-sectional diameter ranged from 0.037 to 0.074 mm. The distance between the saccule and cochlea was around 1 mm. The relation between the RD and SD is shown with micro-CT in Figures 13A,B. The RD runs almost perpendicular against the saccular duct. Figure 13C shows a horizontal section and the UEV. The saccule and cochlear endolymphatic space at the cecum vestibulare are shown in Figure 13D.

## Discussion

### Saccule

The saccule resembled a vertically placed coffee bean with the convex side lying against the spherical recess and the contralateral side facing the vestibule. The superior wall was flattened, lay against the floor of the utricle, and formed an imprint. This suggests that expansion of the saccule may compress the utricle near its macula and vice versa. Basally, the inferior margin of the macula faced the entrance of the RD, where the distance between the otolith membrane and RD was in the region of 0.2–0.3 mm. Here, dislodged otoconia could easily reach the RD by gravity. The saccule wall consisted of a thick and thin region where the thick part lay near the spherical recess. In lower mammals, amphibians, and fish, the saccule contains only the thin part, portentous that the macula may respond to both acoustic and static pressure (26). The orientation of the human saccular macula, the spherical recess and the re-enforced membranes were thought to shield the macula from acoustic oscillations such as stapedius-induced vibrations at large sound levels, in contrast to lower forms. The thin wall appears more flaccid and is probably more prone to dilate and collapse in MD. Ruptures may occur at the junction between the regions by increased pressure due to the resilience. The thin wall of the SD was difficult to image in 3D as it emerged near the band-like reinforcement ~0.9 mm from the utricle floor.

### Utricle and UEV

The utricle is a horizontal cylinder with several extremities. In this study, for the first time, the UEV was viewed three-dimensionally and it appeared as a fantail with a slit-like opening. Its semi-lunar shape differed somewhat from the descriptions by Bast (27) and Anson and Wilson (28). It was located near the floor of the posteromedial wall of the utricle at the internal opening of the VA, as described in humans by Bast (27, 29) and in other mammals by Hoffman and Bast (30). Wilson and Anson (31) described the valve in a 2-year-old child, and they defined it in an adult and named it the “utricular fold.” We used the nomenclature of Perlman and Lindsay (32) for the UD, SD, and ED. They are alleged to form a Y-shaped type I junction in 83% of the cases, with a wide angle between the UD and ED (27). In type II (15%), the UD is short with a broad angle to the SD. In type III (2.8%), there is almost no UD at the right angle to both the SD and ED. We identified a short fan-like UD reaching the UEV with a valve facing the sinus portion of the ED.

There are several theories as to the function of the UEV. Bast (27, 29) suggested that the valve closes the pars superior to regulate endolymph volume. Bast (29) described this as follows (p. 64):

“Its position would indicate that the flow of endolymph is from the endolymphatic duct to the utricle, but this is not a necessary conclusion. If the flow is in the other direction, the slow movement of the endolymph may not affect this valve. On the other hand, in case of sudden pressure disturbance, this valve may prevent the outflow of endolymph from the utricle, thus maintaining a more constant pressure within the utricle and semicircular canals.”

Bast went on to claim that fluid pressure on the valve in the utricle should force it against the opposing duct wall. This was also supported experimentally in the guinea pig, after reducing perilymph and endolymph pressure in the cochlea (33). The valve remained closed, and the utricle and semicircular canals distended even after the collapse of the saccule and cochlear duct. Bast (34) also showed cases of rupture and collapsed saccules, where the utricle was intact. The UD was firmly closed by the UEV. Despite the rupture of the saccule and collapse of the saccule and cochlear duct, the utricle did not collapse. This suggests that endolymph pressure in the utricle can be maintained independently of the pressure in the saccule and cochlear duct. It also proposes that the resolutely closed UEV is responsible for maintaining utricle pressure and therefore acts as a valve. The findings also indicate that fluid is produced in the utricle/semicircular ducts (9). Several regions within the vestibular labyrinth, such as the cupula, have the potential to secrete endolymph similar to the lateral cochlear wall (35, 36). Guild's (5) opinion was that endolymph flows from the utricle and semicircular duct to the ES.

Bast (29) found that in the fetus, the valve is lined by columnar epithelium and at the base with a highly cellular connective tissue that continued into “paralymphatic” tissue around the endolymph system between the utricle and ED. The loose tissue may permit movement of the stiffer valve in case of increased pressure in the utricle. He could not characterize the connective cells but did not exclude the possibility that smooth muscle fibers are present, but there were no indications of nerve fibers. The tissue was independent of the bone surrounding the aperture of the VA. According to Anson and Wilson (28), the fold contains areolar tissue and a fibrous web with a spear-shaped projection of periosteum projecting from the osseous wall but not into the fold proper. This was denied by Bast (27). Anson and Wilson thought that the stiffer valve or lip may not move by pressure changes, while the outer wall could close the orifice and cause movement of fluid in the UD. Schuknecht and Belal (37) studied the valve in humans and found it ideal to protect the pars superior from collapse after dehydration of the rest of the labyrinth. Bachor and Karmody (38) speculated similarly that reduced pressure in the endolymph system, secondary to collapse of the RD, may close the UEV and prevent loss of endolymph from the utricle. Our study showed fibrous connections or pillars between the utricle and the inner surface of the bony labyrinth wall that may stabilize the utricle and also deter it from collapse in cases of reduced endolymph pressure.

Hofman (39) presented 3D imaging of the UEV in laboratory animals using orthogonal-plane fluorescence optical sectioning microscopy and Lim using scanning electron microscopy (40). The valve was flat and funnel-like at the utricle and ran into a narrow and short duct leading to the sinus portion of the ED. Hofman (39) described the valve as fairly rigid. The opposing utricle membrane was thin and appeared to be compliant. They speculated that the outer wall may play a greater role in the opening and closure of the valve.

**Figure 14:**
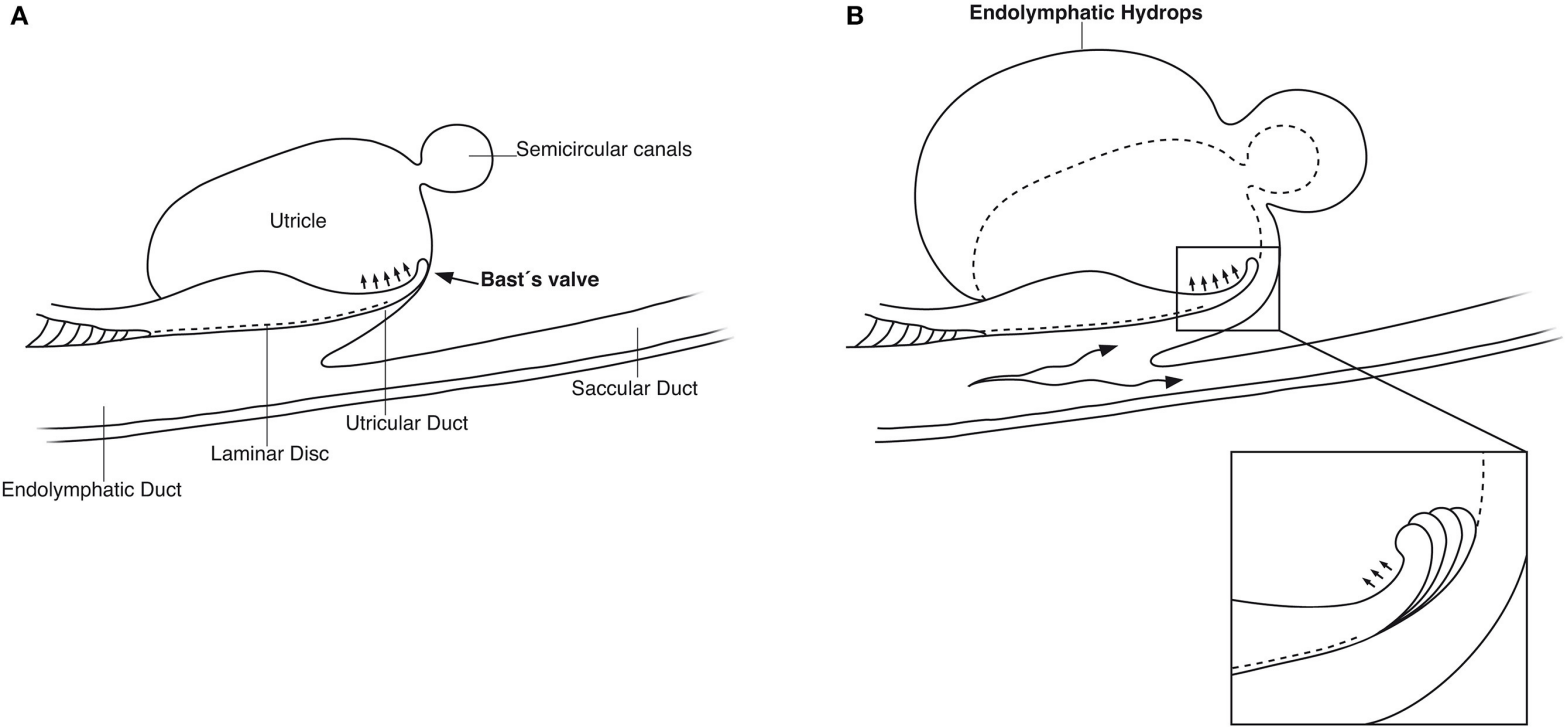
**(A)** Principal drawing of the human utriculo-endolymphatic valve (Bast's valve) based on SR-PCI. The valve is structurally associated with the opening of the vestibular aqueduct through a laminar disc (interrupted line) emerging from the vertical crest of the vestibular aqueduct to the lip of the valve. The outer wall of the utricular duct faces the disc and forms the utricular fold. Between the inner and outer wall at the base of the lip, there is loose tissue allowing the valve to open (small arrows). **(B)** Hypothetical representation of the role of Bast's valve and UD in generating acute endolymphatic hypertension and hydrops in patients with MD. Increased endolymph pressure is caused by reduced reabsorption or hypersecretion in the ES, leading to a “backflow” of endolymph. It compresses the wall of the utricular duct and pushes the valve to open (inset), leading to acute EH and vertigo. Experimentally, secretion in the ES may be provoked within a short timeframe (41, 42).

SR-PCI showed the semi-lunar lip lying against its inner surface with a somewhat different shape, as shown by Bast (27) (Figure 6). We identified reinforcements around the lip after cropping and adjusting the opacity gradient, suggesting that it is fairly rigid (Figure 7). Consequently, increased pressure within the utricle would seem to open the valve through forces acting on the more flaccid part of the opposite membrane. A lowered pressure within the utricle could perhaps close it. External reduction of pressure in the SD and ED could maintain closure to avoid collapse. Conversely, an increased external pressure could force the valve to open by compressing either the flaccid membrane of the utricle or the lip. The UD was found to be partly situated on the loose basal part of the valve, suggesting a mechanism to externally open the lip (Figure 14). A fibrous wall from the VA was also connected to the UD and the valve to hold it in place.

### The RD

The RD was first described by Viktor Hensen in 1863 (4). The Swedish anatomist Gustaf Retzius defined the RD in a newborn in 1884 as a fairly wide channel connecting the cecum vestibulare of the cochlea with the saccule (3). It contrasts with the miniscule channel described histologically by several authors. Its patency has been confirmed at serial sectioning, and both physiological and tracer studies suggest an endolymph flow or communication (5, 18, 41, 43, 44).

Earlier micro-CT did not define the human RD after manual segmentation and contrast enhancement (45). The present micro-CT imaging was able to reproduce it when air entered the vestibule. The small dimension may refute the idea of an ongoing flow under normal conditions. However, size variations may exist, and dilation can be seen after obliteration of the ED (46). Bachor and Karmody described the human RD as small, collapsed, or wide in pediatric temporal bones (38). In half of the bones, the RD was collapsed and seemed closed. They speculated that it may open when pressure increases and permanent closure may lead to hydrops. Since the RD epithelium is stratified and rests on connective tissue enhancement, it is reasonable to believe that the RD can dilate under conditions of increased endolymph pressure and generate a flow. The thin connection may protect the cochlea from leakage of electricity to maintain cell currents at high frequencies. The endolymphatic potential has been found to be low in the vestibule except near the receptors (47, 48).

### EDS in Meniere's Disease

Despite the large number of histopathological studies in MD, only a fraction seem to adhere to the AAO-HNS 1995 criteria. Hallpike and Cairns (49) described two cases where EH was prominent with excessive dilation of the saccule and scala media. There were also changes in the pars superior but no signs of blockage of the communication pathways connecting the utricle, saccule, and ED. The UEV was in its normal position, and the opening of the saccule into the ED contained a dense reddish-staining coagulum in the second case. The absence of loose connective tissue around the ES was a prominent feature. Kyoshiro Yamakawa (50) described similar findings, but there was no mention of the ES. There were edema and calculi in the stria vascularis, the RD seemed open, and the VA was large with colloid-like substance in the duct. He believed that MD symptoms were caused by an augmented pressure in the endolymph which was caused by an increased secretion from the stria vascularis.

EH is a consistent histopathological trait in patients with MD, but its role in the progress of symptoms is unclear. Blockage of the EDS, including the UEV, RD, and SD, is not overrepresented in MD. Dilation of the RD was observed in patients with EH, possibly as a result of increased cochlear hydrostatic pressure (51, 52). Lindsay (53) demonstrated a wide RD in an MD case, but its dimension was not given. According to Shimizu et al. (17) the ED was blocked in 23% in MD, while the UD was blocked in 76% in MD and 52% in normal ears. The SD was blocked in 28% in MD and 76% in normal ears. It suggests that obstruction of the UD and SD may not be the cause of EH in MD. A theory based on CT suggested that displaced saccular otoconia may block the RD, explaining symptoms in MD (54). It is conceivable that a few or even a single displaced otoconia could mechanically obstruct the RD in humans (55). According to Bachor and Karmody (38), there is no correlation between the collapse of RD and cochlear hydrops. Both wide-open and blocked RDs were demonstrated. In patients with MD, the RD was found to have a diameter around 0.1 mm (56). Shimizu (17) showed that the RD in a patient with MD was in the range of 0.05 mm. Kitamura et al. (57) investigated five cases with EH limited to the cochlea with no verified MD. Various obliterations of the saccule and/or RD supported a longitudinal flow of the endolymph. EH depended on the location of the blockage from the RD to the ES. The present study suggests that RD may play a minor role in the exchange of endolymph between the cochlea and vestibule, but under certain conditions, it can dilate and open to maintain cochlear homeostasis.

Schuknecht and Ruther (58) showed that in 42 out of 46 ears, there was either a blockage of longitudinal flow or internal shunts causing fistulae. The UD was blocked in 12, ED in 8, endolymphatic sinus in 9, SD in 7, and RD in 27 bones. They considered that obstructions stopped longitudinal flow from both the pars superior and inferior in 21 cases, only the pars superior in 3 cases, and only the pars inferior in 16 cases. Schuknecht and Belal (37) studied the UEV in 29 human temporal bones with MD and found deposits in the lip and enlargement of the saccule compressing and closing the UD. The 3D reconstructions indicated that the endolymph volume may increase up to three times in MD (59). The relative increase was most prominent in the saccule. A possible explanation is the thin part that may expand even before pressure is built up in the utricle and maintained by the UEV. This could explain MRI findings showing occult or prominent EH preceding symptoms in some patients and in the contralateral asymptomatic ears (60–64).

The role of the ED and ES in MD is still unclear. A diminished absorption in the ED and ES could be due to mechanical obstruction or molecular or fibrotic changes (17) or disturbed vascular drainage (65). Yuen and Schuknecht (66) found no obstruction of the VA in MD and no difference in caliber compared with normal ears. The ED was smaller probably as a result of MD rather than the cause of it. A reduced radiographic visualization of the VA was described by Wilbrand (67) in MD, conceivably explained by its smaller size (68, 69). Results by Monsanto et al. (70) suggested that the UEV and SD may open as a result of retrograde pressure caused by failure of the ES to absorb endolymph. A wide-open valve could disrupt the crista function, resulting in vertigo since it would not protect against pressure fluctuations potentially harmful to the sensory epithelia. An increased pressure in the ED and ES could lead to a reversed flow of endolymph into the utricle and cause selective vestibular disturbance in MD (71).

### Can Acute Endolymph “Backflow” Explain the Meniere Attack?

Experiments have shown that induced alterations in cochlear endolymph volume result in pronounced changes in the ES with bidirectional responses (41, 72). Acute manipulation of the systemic fluid or chronic malfunctions, such as in cochleo-saccular degeneration, modify ES activity (42, 73–76). This suggests that the endolymph moves in either direction between the cochlea and ES and is reabsorbed by the ES under conditions of excess volume and secreted by conditions of volume deficiency. Thus, volume may be regulated in the entire labyrinth by the ES. Potentially, a dysfunction in the ES could lead to a volatile elevation of endolymph pressure and acute cochleo-vestibular symptoms, such as in MD. Nonetheless, there are noticeable differences in the anatomy of laboratory animals. Physiologic adaptation and human upright position may modify fluid exchange and dynamics amid the ear and cranium, with conceivable changes also in the structure of the EDS.

Imaging data suggest that the ED, VA, and UEV form a functional unit for the pars superior. This unit includes a stabilizing fibrous disc projecting from the vertical crest of the internal aperture of the VA to the UD and UEV (Figures 5C, 9C). The UD is directed against and physically associated with the flexible part of the lip of the valve, indicating that the duct may force the valve to open and transmit increased endolymph/pressure into the pars superior (Figure 14). This framework could be particularly prone to mediating sudden escalations of ES pressure into the utricle through the relatively wide UD and UEV. The ES is an expandable bellow-like structure with an extra-osseous part comprising more than two-thirds of the total ES volume. It could mount considerable osmotic pressure gradients against the vestibular labyrinth, despite its 16 times less volume (1.85/30.4 mm^3^) (69, 77, 78). A secretion–degradation system of osmotically active complexes was revealed in the ES acting within a short time frame. Secretion may be triggered by altered volume/pressure conditions in the labyrinth (73), linked to its ability to monitor endolymph (41, 42). A merocrine secretion was already observed in the human ES in an ultrastructural investigation (79). Super-resolution immunohistochemistry recently confirmed a dual absorptive–secretory capacity of the human ES (80). In a temporal bone study in MD, an unproportioned large volume of the ES contained secreted material in an otherwise smaller sac (69).

Therefore, a diminished resorption or hypersecretion of endolymph in the ES could result in a retrograde flow/pressure that underlies the acute attack of vertigo in MD. We speculate, based on the present morphological findings and earlier experimental studies, that acute vestibular symptoms in MD arise through a sudden fluid pressure increase and dilatation caused by a “backflow” of an overcompensated ES secreting into the utricle and semicircular canals. ES endolymph has a unique ionic composition (81), which is potentially noxious to the vestibular receptors. Dilation could lead eventually to membrane ruptures (82) additionally extending the ES response. Pressure may impact saccular and ultimately cochlear functions via the SD and RD with corresponding symptoms. Our results support Seymour's (2) notions that the ES has a supporting secretory role necessary to compensate if volume is reduced to insure complete filling of the phylogenetically older utricle and semicircular canals. Microinjections suggest that elevated hydrostatic pressure in the pars inferior or directly into the utricle result in utricle/vestibular receptor dysfunction (83). These changes were believed to be similar to sudden fluctuating changes in MD suggesting that EH is the cause of the typical symptoms (84).

Our concept could have several clinical consequences. The dysfunction of the ES may arise through hormonal disturbances, stria alterations, immune factors, etc. (85). One proposed way to relieve this dysfunction is through surgical destruction of the ES (86). Nonetheless, more studies of the UEV, EDS, and ES function in MD are necessary and could lead to novel treatment modalities against this troublesome disease. There is also a need for more studies on how these small compartments control fluid pressure and ion homeostasis.

## Data Availability Statement

The raw data supporting the conclusions of this article will be made available by the authors, without undue reservation.

## Author Contributions

GR and JS performed micro-CT on the human cadavers. HL performed image processing. 3D visualization of scanned objects provided by SA, HML, SR, and JS. HR-A was the head of the laboratory and planned the project, analyzed the images, and wrote the manuscript. All authors contributed to the article and approved the submitted version.

## Conflict of Interest

The authors declare that the research was conducted in the absence of any commercial or financial relationships that could be construed as a potential conflict of interest.

## Abbreviations

AAO-HNS: American Association for Head- and Neck Surgery
BM: Basilar membrane
BMIT: Bio-Medical Imaging and Therapy
CLSI: Canadian Light Source, Inc.
CC: Common crus
CT: Computerized tomography
DiceCT: Diffusible iodine-based contrast-enhanced computed tomography
ED: Endolymphatic duct
EDS: Endolymphatic duct system
EH: Endolymphatic hydrops
ES: Endolymphatic sac
LSSC: Lateral semicircular canal
MD: Meniere's disease
Micro-CT: Micro-computerized tomography
MRI: Magnetic Resonance Imaging
PSSC: Posterior semicircular canal
RD: Reunion duct
SD: Saccular duct
SR-PCI: Synchrotron-phase contrast imaging
UD: Utricular duct
UEV: Utriculo-endolymphatic valve (or Bast's valve)
VA: Vestibular aqueduct.
